# The Movement Kinematics and Learning Strategies Associated with Adopting Different Foci of Attention during Both Acquisition and Anxious Performance

**DOI:** 10.3389/fpsyg.2012.00468

**Published:** 2012-11-02

**Authors:** Gavin P. Lawrence, Victoria M. Gottwald, Michael A. Khan, Robin S. S. Kramer

**Affiliations:** ^1^Institute for the Psychology of Elite Performance, School of Sport, Health and Exercise Sciences, Bangor UniversityBangor, UK; ^2^Department of Kinesiology, Faculty of Human Kinetics, University of WindsorWindsor, ON, Canada; ^3^School of Psychology, Bangor UniversityBangor, UK

**Keywords:** attentional focus, performance pressure, novice performers, motor skills, explicit learning

## Abstract

Research suggests that implicit strategies adopted during learning help prevent breakdown of automatic processes and subsequent performance decrements associated with the presence of pressure. According to the Constrained Action Hypothesis, automaticity of movement is promoted when adopting an external focus of attention. The purpose of the current experiment was to investigate if learning with an external focus of attention can enhance performance under subsequent pressure situations through promoting implicit learning and automaticity. Since previous research has generally used outcome measures of performance, the current study adopted measures of movement production. Specifically, we calculated within-subject variability in trajectory velocity and distance traveled every 10% of movement time. This detailed kinematic analysis allowed investigation into some of the previously unexplored mechanisms responsible for the benefits of adopting an external focus of attention. Novice participants performed a 2.5 m golf putt. Following a pre-test, participants were randomly assigned to one of three focus groups (internal, external, control). Participants then completed 400 acquisition trials over two consecutive days before being subjected to both a low anxiety and high anxiety (HA) transfer test. Dependent variables included variability, number of successful putts and mean radial error. Results revealed that variability was greater in the internal compared to the external and control groups. Putting performance revealed that all groups increased performance following acquisition. However, only the control group demonstrated a decrement in performance in the HA transfer test. These findings suggest that adopting an appropriate focus of attention during learning can prevent choking; with an external focus inhibiting the breakdown of automatic processes and an internal focus acting as a self-focus learning strategy and thus desensitizing individuals to anxiety effects.

## Introduction

Previous research suggests that strategies adopted during learning which direct a performer’s focus of attention away from their movements can help alleviate performance decrements typically associated with the presence of pressure (Masters, 1992; Hardy et al., 1996; Jackson et al., 2006). Performing under pressure is an integral part of any athlete’s sporting experience, and an inability to cope with this can often result in an athlete choking, i.e., “performing more poorly than expected, given one’s level of skill” (Beilock and Carr, 2001, p. 701). The current investigation endeavored to inhibit the undesirable effects of choking during anxious performance through manipulating attentional focus during the learning of a golf putt and measuring both movement production and movement outcome.

Atypical performance under pressure has been accounted for with both distraction and self-focus theories. Distraction theories (Eysenck, 1992) suggest that the detrimental effect of anxiety on performance is due to worry consuming the central executive component of working memory, which would normally be used in information processing. Alternatively, self-focus theories (Baumeister, 1984; Lewis and Linder, 1997) suggest that stress can cause performers to become self-conscious and focus on skill mechanics, which can impede performance by disrupting normally automatic response programming and execution. Early research by Fitts and Posner (1967) supports this notion, ascertaining that conscious control is redundant once skills reach the autonomous phase of learning and according to Masters’ (1992) conscious processing hypothesis (CPH) can in fact be detrimental to performance.

Conscious processing hypothesis (Masters, 1992) accounts for the stress performance relationship by suggesting that stress can mediate the reinvestment of conscious control over movements and interfere with normally automatic response programming, thus disrupting performance. Masters reasons, that under pressure, self-consciousness initiates the breakdown of larger, integrated chunks of information into separate, smaller units, thus prolonging information processing. Furthermore, Masters proposes that if automatic processes are encouraged during learning, through the development of implicit knowledge, then reinvestment of conscious explicit knowledge cannot occur and consequently the breakdown of information units as associated with the presence of anxiety is less likely to transpire. Previous research exploring ways of preventing this breakdown in automatic processes, as a means of maintaining performance under pressure, advocates analogy learning as a successful method of reducing acquisition of explicit knowledge (Masters, 1992). Holistic imagery, holistic process goals, and modeling are advocated as ways to encourage automatic processes, through the development of conceptual representations of skills during learning (Hardy et al., 1996).

Similarly, the Constrained Action Hypothesis (CAH; Wulf et al., 2001) suggests the adoption of external focus of attention as an alternative strategy to reduce the breakdown of automatic processes. This involves directing attention to movement effects such as the swinging motion of the club in golf, as opposed to directing focus to body movements, such as the swinging motion of the arms (internal focus of attention). Wulf (2007) suggest that focusing on movements themselves, as opposed to movement effects, reduces the congruence between planning and action and disrupts the usually automatic control process. Focusing on the effects of one’s movement by adopting an external focus, prevents this process, and serves to enhance performance (Wulf et al., 1998). Support for this hypothesis has been demonstrated across a variety of domains and populations (for a review, see Wulf, 2007).

However, preceding research has typically adopted outcome measures of performance such as speed and accuracy and thus neglected to investigate what specific adjustments may occur in movement production as a result of adopting differing foci of attention (Wulf, 2007; Lawrence et al., 2011). One study which has examined performance kinematics under internal and external focus conditions is that of Lohse et al. (2010). Here researchers revealed that shoulder variability at the point of release in a dart throw was greater under an external compared to internal focus. Since findings also revealed that the accuracy of the dart throws were greater in the external compared to internal group, the researchers suggested that the effect was due to “functional variability” (see Müller and Loosch, 1999). The suggestion here is that when a motor skill is performed repeatedly over time, even to the point of expertise, clear deviations in movement kinematics occur regardless of what becomes an extremely consistent movement outcome. Since performance is consistently high, the kinematic variability during movement execution is described as functional as it facilitates consistent and accurate movement outcomes. This concept is consistent with the work of Bernstein (1967) who studied the number of different ways a unit of control (for example joints, muscles, motor units) is capable of moving, i.e., “degrees of freedom.” These degrees of freedom are thought to freeze and release depending on what stage of learning a performer has reached. For example, in early cognitive stages of learning Fitts and Posner (1967) observe how movements are often rigid and constrained, where degrees of freedom are frozen. Through extensive practice performers are able to release degrees of freedom, resulting in more fluid and effective movement execution as observed in the Associative stage of learning. Once the performer reaches the Autonomous stage of learning, degrees of freedom are once again re-organized for optimal performance. This economizing of degrees of freedom in both early and late phases of learning serves two different functions. During novice performance, degrees of freedom are highly constrained because learners are simply unable to control too many reactive forces. This is in contrast to experts who constrain degrees of freedom in order to ensure muscles, joints, and motor units function as an effective and efficient synergy to produce optimal performance (for a review see Rose and Christina, 2006). The current study investigated the effect of differing foci of attention of movement variability further by utilizing Vicon motion analysis together with a novel methodology previously designed to investigate the role of vision in adjusting limb trajectories in upper body target-directed aiming movements (for a review see Khan et al., 2006). This method allows researchers to examine the variability in both distance traveled and velocity at various percentiles of overall movement time. In line with Lohse et al. (2010) and Müller and Loosch’s (1999) notion of functional variability, it was hypothesized that participants who adopted an external focus of attention during learning would exhibit more consistent movements than those who learned under an internal focus of attention condition.

Current focus of attention literature (Wulf, 2007) would suggest that attaining explicit knowledge regarding movement production (i.e., internal information), would be more detrimental to performance comparative to explicit knowledge regarding movement effects (i.e., external information). As such, a secondary aim of the current study was to investigate this further in an attempt to understand if it is the acquisition of explicit knowledge in general, which governs anxious performance or whether it is a specific *type* of explicit knowledge (i.e., explicit knowledge regarding ones movements or the effects of one’s movements on the environment). To achieve this participants learnt a golf putting task under either internal, external, or control conditions and were then subjected to an anxious performance condition. If the choking phenomenon is reduced in the external compared to internal focus of attention condition then one can propose that reinvestment in explicit knowledge is only detrimental to performance if that knowledge is centered around movement mechanics.

Beilock and Carr (2001) analyzed attention during skill execution by examining the quantity of episodic memories for both experts and novices and revealed that automaticity of expert performance results in fewer episodic memories. In addition, Poolton et al. (2006) suggested that participants who adopt an internal focus of attention tend to acquire more internal rules regarding their movements. Thus, it was expected that those participants who learned under an external focus of attention conditions would develop fewer explicit rules regarding the movement itself (internal rules) compared to those who adopted an internal focus of attention and would thus be less likely to choke under pressure as breakdowns in automatic programming would not occur in the absence of these rules.

If we consider the underlying theoretical foundations which underpin both the CAH and CPH, it is surprising that researchers have not previously considered the two lines of research in conjunction to a greater extent prior to this investigation. A principle study by Bell and Hardy (2009), which did examine the focus of attention literature in line with anxiety literature revealed that expert performance of golf chipping was enhanced by an external focus of attention under both low and high anxiety conditions. The current research elected to adopt a learning paradigm to investigate the effects of anxiety on golf putting performance when participants have acquired a skill under different attentional foci conditions. It was hypothesized that learning under an external focus of attention would lead to more permanent (learnt) behaviors that promote action production and execution without movement centered knowledge, which would result in consistent performance between low and high anxious situations (i.e., eliminate the choking phenomenon).

The aim of the current investigation was to fill some of the more pertinent gaps in the focus of attention and anxiety literature. Specifically, to examine if explicit learning (regarding movements) can be minimized through adopting an external focus of attention during learning and thus prevent performance breakdown under subsequent anxious conditions. Additionally, a novel variability methodology was utilized to investigate the effects of different foci of attention on the entire movement trajectory. Thus, allowing detailed investigation into the motor production mechanisms that might account for the outcome performance differences associated with adopting different foci of attention. To achieve these aims, performance (as measured by both movement production and movement outcome) was investigated whilst participants learnt a golf putting task under either an external focus of attention, an internal focus of attention or control (no focus), and when participants were subsequently transferred to both a low anxiety (LA) and high anxiety (HA) transfer tests.

## Materials and Methods

### Participants

Twenty nine participants mean age 22.1 years (SD = 4.3); mean mass 69.3 kg (SD = 13.0); mean height 171.2 cm (SD = 8.6) with no previous experience in golf, volunteered to participate^1^. All were naïve to the research hypotheses being tested and gave their informed consent prior to taking part in the investigation. The experiment was conducted in accordance with the institutions ethical guidelines for research involving human participants.

### Task and apparatus

**Figure 1:**
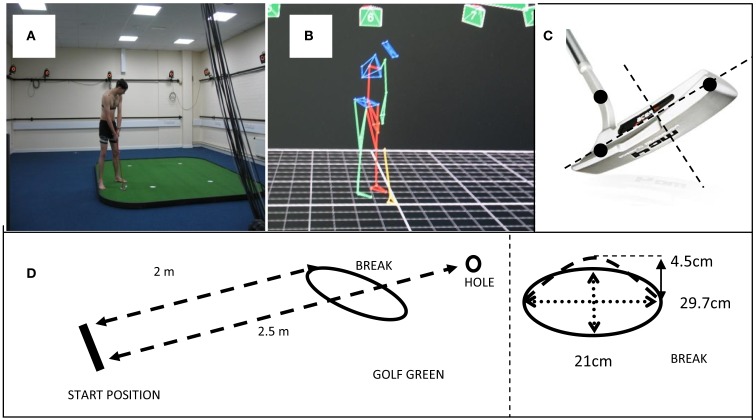
**(A)** Twelve camera Vicon system and putting surface; **(B)** example screen shot of Nexus software; **(C)** retro-reflective marker placement on club-head; **(D)** schematic of the putting set up and breaker placement.

The task was a 2.5 m golf putt performed on a Huxley Premier Pro turf putting green (8′ × 12′). The green was set up with a standard “Huxley incliner” placed under the surface of the green 1 m from the start and positioned just left of the line of the putt. This created a convex half-sphere with an incline rising to 4.5 cm resulting in a left to right breaking putt (see Figure 1D). Participants putted toward a 10.5 cm hole, consistent with standardized PGA requirements, using a standard KT25 Prosimmon golf putter and Slazenger Raw Distance 432 dimple pattern golf ball. A 12 camera Vicon Nexus system (see Figures 1A,B) sampling at 100 Hz was used to track co-ordinates of three retro-reflective markers (14 mm diameter). Two markers were placed on the head of the golf club and the final marker was placed at the lower end of the shaft (see Figure 1C). The Vicon Nexus system utilized marker movements in the *XYZ* plane in order to calculate and plot movements in 3D space. A heart rate monitor (Polar T31 chest strap model) was placed on the participant and HR (bpm) data was recorded.

### Procedure

At the start of testing, participants were informed that the purpose of the investigation was to examine the accuracy of golf putting over a period of practice trials. It was explained that the goal of the task was to putt the ball as accurately as possible and that task performance would be assessed by both the number of successful putts (NSP) and the distance from the hole the ball terminated on unsuccessful putts. Participants completed a 25-trial pre-test before being randomized into either an internal focus of attention group (*N* = 9), an external focus of attention group (*N* = 10), or a control group (*N* = 10). Participants in the internal focus group were instructed to putt whilst simultaneously focusing on the “swinging motion of their arms,” those in the external focus group were instructed to focus on the “swinging motion of the golf club” whilst those in the control group were given no focus of attention instructions. Focus instructions were adopted from Wulf and Su (2007) and directed participants on “what” to attend to but not “where” to look. Participants performed 400 acquisition trials which were split into four blocks of 50 trials and performed over two consecutive days (blocks one and two on day one and blocks three and four on day two). For all participants, their relevant attentional focus instructions were clearly posted on the laboratory room wall and provided verbally at the start and mid-point of every block of trials. A simple focus question, devised according to each group, was asked following every trial and served to reinforce focus reminders (see Lawrence et al., 2011).

At the end of the acquisition trials participants were asked to leave the testing room and given a short break. Following this, participants completed 25 trials under LA before finally completing a further 25 trials under HA. Participants were not given any attentional focus instructions or reminders during the transfer tests. After completing the HA test participants reported their episodic memories. Specifically, participants were asked to describe the last putt they had taken in enough detail so that a friend would be able to replicate it (adopted from Beilock and Carr, 2001).

A single (combined) post manipulation questionnaire was carried out at the end of day two following both transfer conditions^2^. Participants were asked to state the intensity of their focus on internal and external foci, revealing the extent to which they were able to adopt the appropriate focus of attention and also to what extent they adopted an inappropriate focus of attention.

### Anxiety manipulation

Anxiety was manipulated by a combination of ego threat and social evaluation, which has previously been utilized successfully (Masters, 1992; Beilock et al., 2002). Specifically, participants were asked to leave the room for a short duration, and informed that during this time their performance would be discussed by the two researchers present during testing. Upon their return participants were informed that they had been paired randomly with another participant and that if they both improved their putting performance by 20% then they would both receive £10. They were then informed that their partner had already improved their performance by the required 20% and thus they would need to do the same in order for both them and their partner to receive the money. In addition, they were told “if you fail to improve your performance by the required 20%, your partner will be informed of your identity and told that you had been unsuccessful and thus neither of you can now receive the prize money.” Finally, participants were informed that this final block of trials would be video recorded and then sent off to an expert golfer for analysis and that if they missed 75% or more of their putts their name would be added to a loser-board, posted around the sports science department.

### Dependent measures and analyses

#### Performance data

The primary measure of performance was the NSP and the secondary, less dichotomous measure of outcome performance was Mean Radial Error (MRE). MRE was calculated as the distance from the ball to the hole, using the following formula:

$${\left(x^{2}+y^{2}\right)}^{0.5}$$

where *x* is the distance from the hole perpendicular to the direction of the putt and *y* the distance from the hole in the same direction of the putt.

To ensure there were no significant differences between the performance of groups prior to testing, the means of pre-test NSP and MRE performance data were submitted to separate one way (group) ANOVAs. In order to assess performance from pre-test to transfer separate 3 group [control (Con), internal (Int), external (Ext) × 3 block pre-test (Pre), LA, HA] mixed model ANOVAs were performed on the mean NSP and MRE data. Significant between-subject effects were broken down using Tukeys HSD *post hoc* tests while significant within-subject effects were broken down into their simple main effects.

#### Episodic memory

Measurements of episodic memory were taken to determine both the number of explicit knowledge/rules acquired, as well as the qualitative content of this knowledge. A one way ANOVA was performed on the total number of episodic memories for each group. To analyze the qualitative content, memories were subjectively categorized as being either internal (e.g., swinging of the arms; kept arms straight) or external (e.g., lining the club up with the ball and hole; swinging of the club). This categorization was performed by two independent researchers and a 3 group (Con, Int, Ext) × 2 type (internal/external) ANOVA was then performed on this data.

#### Motion analysis and variability

Vicon motion analysis was used to calculate co-ordinates of markers in the *x*, *y*, and *z* planes of movement. Researchers then calculated both velocity and distance traveled every 10th percentile of each trial’s movement time. The within-subject SD of this data was then calculated and the means of this data provided a measure of variability throughout movement trajectory. This process was completed for both the back-swing and forward-swing of the golf putt up until ball contact and then again for the swing post ball contact (i.e., the follow through of the club). Thus, we calculated separate measures of variability in both velocity and distance traveled every 10% of the movement time for these three putting phases. To analyze the data, separate 3 group (Con, Int, Ext) × 3 experimental phase (Pre, LA, HA) × 10 position (10, 20, …, 100%) mixed model ANOVAs were performed on resultant *XYZ* data for both velocity and distance traveled; these analyses were conducted for each of the three previously described putting phases resulting in a total of six 3 group × 3 experimental phase × 10 position mixed model ANOVAs.

#### Anxiety data

The Mental Readiness Form-3 (MRF-3; Krane, 1994) was used to determine competitive anxiety and was administered prior to both LA transfer and HA transfer conditions. Additionally, HR gave a physiological indicator of anxiety and was recorded throughout LA and HA transfer conditions. This data was submitted to a three group (Con, Int, Ext) × 2 experimental phase (LA, HA) ANOVA.

## Results

### Anxiety (MRF-3 and HR)

Results of the MRF-3 data revealed a significant main effect for experimental phase (*F*_1,26_ = 19.35, *p* < 0.01, η^2^ = 0.43) with anxiety scores significantly increasing from LA to HA transfer (LA = 10.03, SD = 4.14; HA = 13.76, SD = 6.63). The results of the self-reported anxiety scores were supported by the HR data, which revealed a significant main effect for experimental phase (*F*_1,26_ = 9.68, *p* < 0.01, η^2^ = 0.27) with a significant increase in HR from LA to HA transfer (LA = 83.95, SD = 9.57; HA = 86.66, SD = 10.76). For both variables, main effects for group and group × experimental phase interactions were non-significant (*p* > 0.05).

### Performance (NSP)

#### Pre-test

Results at pre-test revealed no significant difference between the NSP of participants in different conditions (*F*_2,26_ = 3.24, *p* > 0.05; internal = 3.67, SD = 0.83; external = 2.70, SD = 1.34; control = 4.70, SD = 1.25). Thus, any performance differences cannot be attributed to differences prior to the investigation.

#### Pre-test, LA, and HA

**Figure 2:**
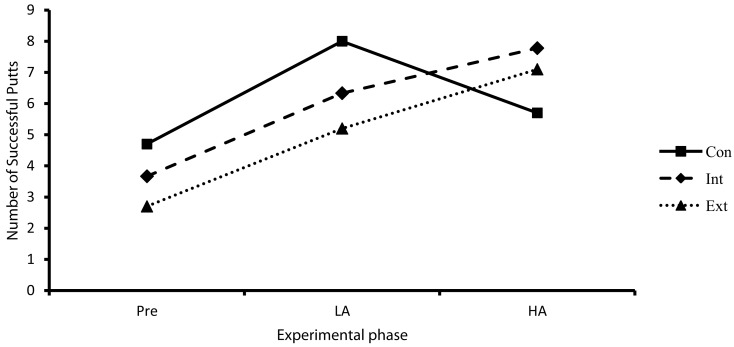
**Performance (NSP) as a function of attentional focus (Con, control; Int, internal; Ext, external) and experimental phase (Pre, pre-test; LA, low anxiety; HA, high anxiety)**.

Results revealed a significant main effect for experimental phase (*F*_2,52_ = 16.45, *p* < 0.01, η^2^ = 0.39) and a significant group × experimental phase interaction (*F*_4,52_ = 2.80, *p* < 0.05, η^2^ = 0.18). Breakdown of this revealed a significant decrease in NSP from LA to HA for participants in the control group, a significant increase in NSP from LA to HA for participants in the external focus group and no change in NSP from LA to HA for participants in the internal focus group (see Figure 2).

### Performance (MRE)

#### Pre-test

Results at pre-test revealed no significant difference between the NSP of participants in different conditions (*F*_2,26_ = 0.05, *p* > 0.05; internal = 601.67, SD = 169.98; external = 575.33, SD = 130.13; control = 586.11, SD = 203.20). Thus, any performance differences cannot be attributed to differences prior to the investigation.

#### Pre-test, LA, and HA

**Figure 3:**
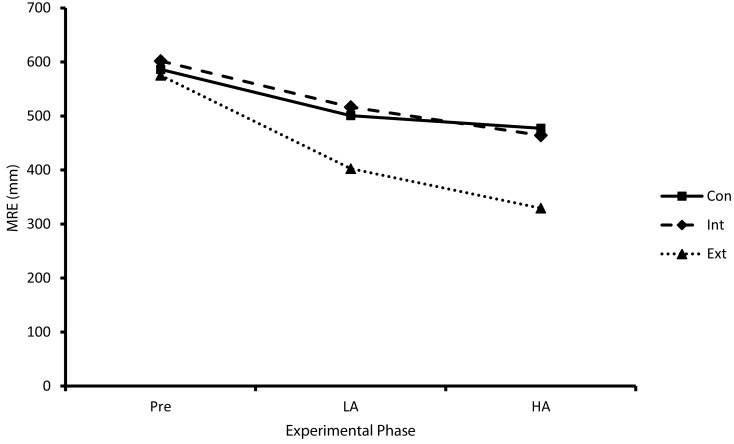
**Performance (MRE) as a function of attentional focus (Con, control; Int, internal; Ext, external) and experimental phase (Pre, pre-test; LA, low anxiety; HA, high anxiety)**.

Results revealed significant main effects for experimental phase (*F*_1.65,42.81_ = 33.34, *p* < 0.01, η^2^ = 0.56) and group (*F*_2,26_ = 3.38, *p* < 0.05, η^2^ = 0.21) but no significant group × experimental phase interaction (*F*_4,52_ = 0.97, *p* = 0.43, η^2^ = 0.07). Specifically, MRE significantly decreased as a function of experimental phase and the control group exhibited significantly greater MRE comparative to the external focus group (see Figure 3).

### Episodic memory: Total number and type of memories/rules

Details of the both the number and type of memories reported are presented in Table 1. Results for the analysis of number of memories reported revealed no significant group differences (*F*_2,28_ = 0.40, *p* > 0.05; mean rules; Control = 4.1, SD = 3.21; Internal = 5.2, SD = 2.10; External = 4.3, SD = 2.87).

**Table 1:** **Episodic memories reported; categorized as internal or external**.

Internal	External
Feet shoulder width apart	Looked at the ball
Bent knees	Looked at the hole
Leant forward	Lined up putter with target
Right hand below left	Putter head square to target
Wrist action	Slowly pulled putter back
Straight arms	Swing of the club
Pendulum motion of arms	Followed through the ball

Results of the analysis on the type of memories revealed a significant group × type interaction (*F*_2,27_ = 5.01, *p* < 0.05, η^2^ = 0.27). Breakdown of which confirmed that participants in the internal focus of attention group acquired a significantly higher number of internal memories than participants in the control and external focus of attention groups. Similarly, participants in the external focus of attention group acquired a significantly higher number of external memories compared to participants in the control and internal focus of attention groups. There was no difference between the amount of internal and external memories reported by the control group.

### Variability pre ball contact

#### Distance traveled *XYZ*

##### Back-swing

**Figure 4:**
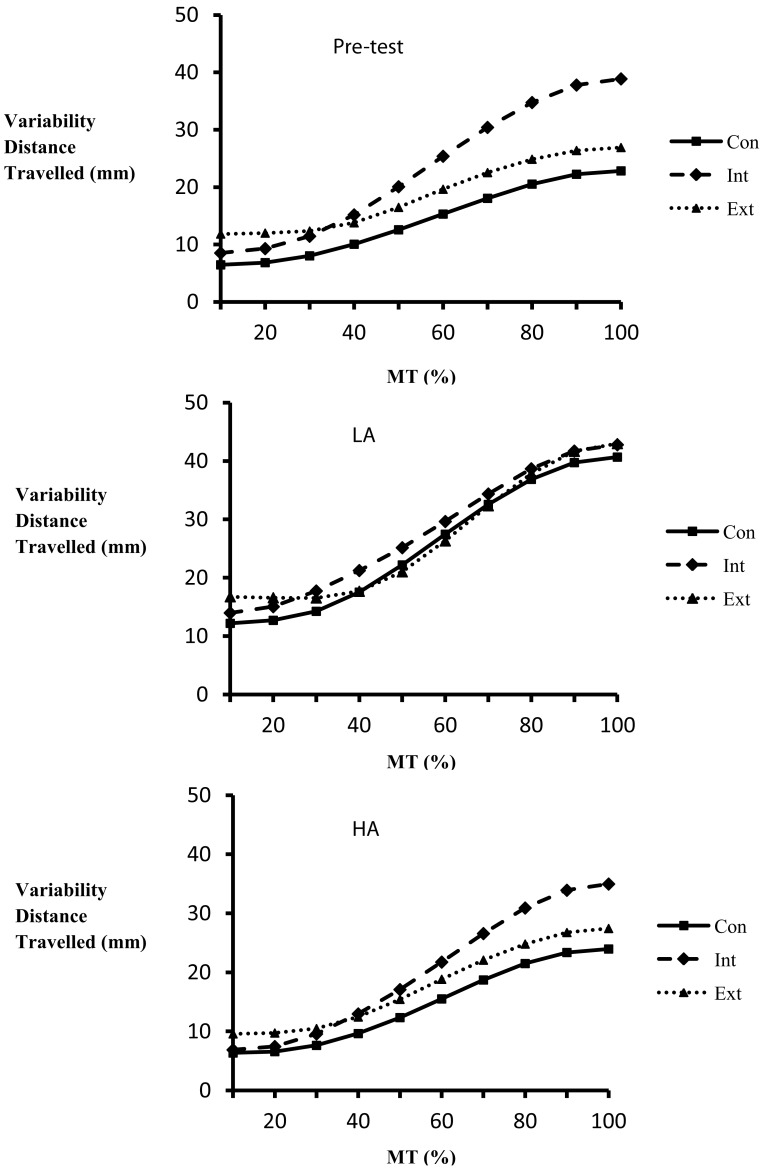
**Variability in distance traveled at every 10% of MT for the back-swing as a function of group (Con, control; Int, internal; Ext, external) and experimental phase (Pre, pre-test; LA, low anxiety; HA, high anxiety)**.

Results revealed a significant main effect for experimental phase (*F*_2,52_ = 22.70, *p* < 0.01, η^2^ = 0.47), position (%; *F*_9,234_ = 269.81, *p* < 0.01, η = 0.91), and group (*F*_2,26_ = 4.05, *p* < 0.05, η^2^ = 0.24). There were also experimental phase × position (*F*_18,468_ = 5.32, *p* < 0.01, η^2^ = 0.17), position × group (*F*_18,234_ = 3.72, *p* < 0.01, η^2^ = 0.22), and experimental phase × position × group interactions (*F*_36,468_ = 1.64, *p* < 0.05, η^2^ = 0.11). Specifically, breakdowns confirmed that variability was greater during the pre-test, and more so during the second half of the back-swing. This increase in variability throughout the back-swing was significantly greater for the internal group compared to the external and control group with this effect being greater in pre-test and HA compared to the LA (see Figure 4).

##### Forward-swing

Results revealed a significant main effect for experimental phase (*F*_2,52_ = 19.65, *p* < 0.01, η^2^ = 0.43), with variability significantly decreasing from pre-test to LA and from pre-test to HA, a main effect for position (*F*_9,234_ = 518.37, *p* < 0.01, η^2^ = 0.95), with variability significantly decreasing as a function of MT percentage, and a main effect for group (*F*_2,26_ = 4.89, *p* < 0.05, η^2^ = 0.27), with the internal group being significantly more variable than the control group. Additionally there were significant experimental phase × position (*F*_18,468_ = 13.69, *p* < 0.01, η^2^ = 0.35) and position × group interactions (*F*_18,234_ = 4.38, *p* < 0.01, η^2^ = 0.25). Breakdowns confirmed that pre-test variability was greatest at the start of the forward-swing and that this effect was significantly greater for the internal group compared to the external and control groups.

#### Velocity *XYZ*

##### Back-swing

Results revealed a significant main effect for experimental phase (*F*_2,52_ = 31.53, *p* < 0.01, η^2^ = 0.56) with variability decreasing from pre-test to LA and again from LA to HA and a main effect for position (*F*_9,234_ = 191.47, *p* < 0.01, η^2^ = 0.88) with variability increasing during the first 50% of the back-swing before decreasing during the second 50% of the back-swing. Additionally there were significant experimental phase × position (*F*_18,468_ = 18.43, *p* < 0.01, η^2^ = 0.42) and experimental phase × position × group interactions (*F*_36,468_ = 2.06, *p* < 0.01, η^2^ = 0.14). Breakdowns revealed that variability was significantly greater at the mid-point of the back-swing during the pre-test and that this effect was significantly greater for the internal and external groups compared to the control group.

##### Forward-swing

Results revealed a significant main effect for experimental phase (*F*_2,52_ = 60.36, *p* < 0.01, η^2^ = 0.70) and position (*F*_9,234_ = 250.81, *p* < 0.01, η^2^ = 0.91), together with significant experimental phase × position (*F*_18,468_ = 26.31, *p* < 0.01, η^2^ = 0.50) and experimental phase × group interactions (*F*_4,52_ = 3.00, *p* < 0.05, η^2^ = 0.19). Breakdowns confirmed that variability was greatest during pre-test for the second half of the forward-swing and that the external group was significantly more variable than either the control or internal group at pre-test.

### Variability post ball contact

#### MT *XYZ*

Results revealed a significant main effect for position (*F*_9,243_ = 109.39, *p* < 0.01, η^2^ = 0.80). There were also significant experimental phase × position (*F*_18,486_ = 5.00, *p* < 0.01, η^2^ = 0.16) and position × group interactions (*F*_18,243_ = 3.21, *p* < 0.01, η^2^ = 0.19). Specifically, variability significantly increased throughout the forward-swing with this effect being significantly more pronounced during the pre-test compared to either transfer test and for the internal group compared to both the external and control group.

#### Velocity *XYZ*

Results revealed significant main effects for experimental phase (*F*_2,54_ = 17.62, *p* < 0.01, η^2^ = 0.40) and position (*F*_9,243_ = 81.78, *p* < 0.01, η^2^ = 0.75). There were also significant experimental phase × group (*F*_4,54_ = 3.87, *p* < 0.01, η^2^ = 0.22), experimental phase × position (*F*_18,486_ = 15.97, *p* < 0.01, η^2^ = 0.37), and experimental phase × position × group interactions (*F*_36,486_ = 2.50, *p* < 0.01, η^2^ = 0.16). Breakdowns revealed a decreasing variability throughout the forward-swing and over time. Furthermore, variability of the control group was significantly more pronounced than either the internal or external groups at pre-test during the first half of the forward-swing.

## Discussion

The purpose of the current study was three fold; 1, to investigate the effects of learning under different attentional focus conditions on subsequent anxious performance; 2, to determine if the positive effects of learning with an external focus of attention on anxious performance could be explained by the nature of the explicit rules generated during learning; 3, to adopt dependent measures that allowed investigation into the effects of the different attentional foci on movement kinematics (a previously neglected performance variable within the attentional focus literature). Findings revealed that participants who adopted an external focus of attention throughout acquisition continued to improve performance (NSP) when subjected to pressure whereas participants devoid of focus instructions displayed classic choking behaviors. The results surrounding the explicit rules generated during learning suggested that the protective effect of practicing under an external focus of attention can be explained, in part, by the development of explicit skill rather than movement centered rules. Finally, the above effects were not represented clearly within the movement kinematics of the learnt action, since results of the variability profiles for both velocity and distance traveled during all phases of the golf putt (back-swing, forward-swing to ball contact and forward-swing post ball contact) did not clearly account for why the control group demonstrated performance decrements under pressure and the internal and external focus groups did not.

There is a considerable body of evidence to suggest that adopting differential attentional foci results in performance differences at retention (Wulf et al., 1999, 2002; McNevin et al., 2003). However, results of the current investigation revealed no performance differences following acquisition trials. Nevertheless it is plausible that there may have been discrepancies in performance earlier in the acquisition process. For example, previous research revealing acquisition differences between internal, external, and control foci of attention conditions have been conducted with notably fewer acquisition trials (e.g., Wulf and Su, 2007 incorporated just 60 acquisition trials in a novice golf putting task), thus it is reasonable to suggest that these differences may have been extinguished after the 400 acquisition trials adopted in the present investigation. Indeed, Poolton et al. (2006) incorporated a substantial 300 acquisition trial protocol and, similar to that of the current investigation, found no performance differences at retention.

Despite the null findings in retention, differences in performance were certainly evident once pressure was induced. Although relatively low compared to genuine competition anxiety, the anxiety manipulation was successful in raising both self-reported anxiety scores (MRF-3) and HR, between the LA and HA transfer. Consistent with CPH (Masters, 1992) the control group’s performance deteriorated as a consequence of elevated performance pressure. CPH accounts for this by proposing that anxiety instigates the reinvestment of conscious control over actions, which inhibits normally automatic response programming and thus impedes performance. Hence, performance of the control group deteriorated to a level similar to early in learning.

However, when adopting an external focus of attention this negative effect of anxiety on performance was negated. Whilst these findings are consistent with research by Bell and Hardy (2009), who illustrated the benefits of an external focus of attention on expert golf chipping performance under pressure, the theoretical explanations offered for the current findings are different. The rationale for this is twofold, firstly, the skill levels of participants and consequential performance or learning paradigms adopted by both investigations are very different: Bell and Hardy utilized expert performers and a performance paradigm whereas the current investigation utilized novice participants and a learning paradigm. Secondly, only the current investigation included a measure to investigate explicit rule generation during skill execution. The exclusion of this in Bell and Hardy’s research meant that the CPH and the theory of reinvestment (Masters, 1992) could not be fully investigated as a plausible explanation for their findings. Consequently, Bell and Hardy account for their findings with the distraction hypothesis (Wine, 1971). They suggested that since skill execution consumes less attentional capacity under an external focus of attention comparative to an internal focus of attention (Wulf et al., 2001), the attentional threshold is less likely to be exceeded when anxiety is present and thus performance is maintained or even enhanced. If one looks at performance data alone, distraction is a plausible explanation for the findings of the current investigation. However, if one looks at these in conjunction with the results surrounding the amount and type of explicit rules utilized during transfer, it is more likely that benefits of learning with an external focus of attention on subsequent anxious performance are due to a learning strategy which prevented reinvestment. Poolton et al. (2006) previously revealed that participants adopting an external focus of attention accumulate significantly fewer rules regarding their movements comparative to those adopting an internal focus of attention. The results of the current investigation supported these findings, revealing a significant difference in the nature but not the number of explicit rules developed during learning under either the internal or external focus of attention groups. Specifically, the external focus of attention group reported less explicit knowledge regarding movements of their body than both the internal focus of attention and control groups. As such, when placed into the HA transfer test the golf putting skill of these participants were less likely to breakdown as explicit knowledge regarding skill movement was reduced (Masters, 1992). It should be noted here that whilst the external focus of attention group reported less explicit knowledge surrounding the mechanics of their movements compared to the internal focus of attention or control group, they did not actually report generating fewer explicit rules during learning. These results are in line with our second hypothesis suggesting that it is the *type* and not the *number* of explicit rules performers generate that govern reinvestment under anxious conditions. Consequently, reinvestment theory should be extended to clarify that tasks are more likely to break down under anxiety if performers have accumulated accessible and conscious task-relevant knowledge that is centered around body movements (i.e., the swing of the arms) and not necessarily around skill movements (i.e., the swing of the club).

Contrary to expectations, participants adopting internal focus instructions during learning were able to maintain performance under pressure. According to the CPH (Masters, 1992) the internal focus of attention group should have invested explicit movement knowledge under pressure, resulting in performance decrements. However, previous research by Beilock and Carr (2001) determined that self-consciousness training can protect against the debilitative effects of choking under pressure. Beilock and Carr had participants practice golf putting under self-focus conditions and revealed that when these participants were subjected to an anxiety test they were able to maintain performance, suggesting that training under conditions of self-consciousness can lead to a reduction in the choking phenomenon. Similarly, in the current study, participants adopted an internal focus of attention during learning and so became familiarized with consciously controlling movements and thus it is possible that the self-focus induced by the presence of anxiety did not disrupt performance since participants were accustomed to performing under these conditions. This notion is consistent with Henry’s (1968) specificity of learning principle, whereby practice conditions that most closely approximate the movements of the target skill and the environmental conditions of the target context result in the best learning experiences. Thus, if anxiety induces a self-focus then learning conditions that also prompt a self-focus or internal focus should facilitate optimal performance in anxiety inducing situations.

In order to further investigate the effects of different foci of attention on both LA and HA performance, we examined the variability of both the velocity and the distance traveled throughout the movement trajectory. This allowed for insights into functional variability (Müller and Loosch, 1999) and how external and internal foci of attention affect the kinematics of movements, something that until the current investigation has received little research attention. In line with Lohse et al. (2010) it was expected that movements in the external focus of attention group would be more variable and result in greater outcome accuracy (i.e., participants would be releasing degrees of freedom with the function of allowing exploration of the perceptual-motor workspace to achieve the most effective and accurate trajectories), than those in the internal focus of attention group. The rationale was that adopting an internal focus of attention would constrain normally automatic movement control leading to less effective actions and lower movement variability compared to situations where an external FOA is adopted. Furthermore, in line with the CAH (Wulf et al., 2001) and the CPH (Masters, 1992) it was expected that both the internal focus of attention and control groups would suffer a similar constraining of the action when participants were subjected to the anxiety transfer test. That is, through the adoption of either a self-focus or through the reinvestment of explicit movement centered knowledge, normally automatic or proceduralized skills would be constrained and/or broken down into step by step processes. This over-analysis and breakdown of automatic movement would manifest itself in ineffective actions and result in reduced kinematic variability.

The analysis of the variability profiles for both the velocity and the distance traveled data only revealed significant group differences at the LA and HA transfer tests in the distance traveled during the back-swing of the golf putt. Specifically, variability was greater in the internal focus of attention group compared to both the external and control groups at the LA and HA tests. Whilst this finding is not supportive of the above hypothesis, the authors suggest that the increase in variability is because the internal focus of attention encourages participants to over analyze movements which resulted in maladaptive corrections that produced trial to trial variability. That is, participants were making both online (i.e., during action) and offline (i.e., between trials) adjustments to movements as a result of focusing solely on the internal aspects of the movement during action (see Lai and Shea, 1999). However, the NSP data does not suggest that this increase in variability during the back-swing of the movement trajectory was sufficient to alter overall performance. Indeed there were no group differences in NSP at retention (LA test) and only the control group experienced a decrease in putting performance during the HA test despite not demonstrating any alterations in the variability of the movement trajectory. Although the internal focus of attention group increased variability it is possible that the null effect of this on putting performance was as a result of the previous discussed specificity of learning principle. That is, training under the self-focus condition resulted in a development of a variable action that at the end of 400 acquisition trials was able to effectively meet the requirements of the task goal. When, conditions then changed to include anxiety the effect this emotional state had on the control of movement was similar to that of training (i.e., movements were performed under self-focus conditions in both acquisition and anxiety transfer). Thus, both the movement trajectory and movement outcome performance was comparable between the acquisition and the HA transfer conditions. In addition, it seems likely that the adopted markers of variability (i.e., variability in the back-swing and forward-swing of the golf club-head) of the current investigation may not be the most pertinent predictor of outcome performance. For example, Mullen (2000) revealed that wrist kinematics significantly predicted outcome performance in a high anxiety situation. Specifically, individuals that experienced a decrement in performance as a result of anxiety displayed reduced variability at the wrist compared to those who maintained performance levels under anxiety. Thus, in the current investigation, it is possible that the movement variability of the internal focus of attention group was constrained outside the movement of the golf club. Future research should investigate this possibility further by exploring variability throughout the action over the entire body.

One possibility to account for the non-significant change in the variability of the control group between the LA and HA transfer, despite the decrement in NSP, may be that trajectories were consistently inaccurate in terms of the line of the putt when under pressure. This proposal would account for why the variability profiles between the control and external focus groups did not differ at the HA transfer test. That is, whilst the external focus of attention group produced consistent putts of an accurate nature, the control group produced consistent putting actions with an inaccurate outcome. This would result in variability remaining relatively stable regardless of a decline in performance between LA and HA retention.

In conclusion, the present investigation demonstrated that adopting an external focus of attention during learning is beneficial for maintaining performance under subsequent anxiety conditions. We propose that the protective effect of acquiring skills under an external focus of attention is due to the nature of the explicit knowledge developed under these conditions not being centered around the mechanics of body movements. In addition and contrary to expectations, it appears that learning under an internal focus of attention also prevents decrements in performance typically associated with the presence of anxiety. We suggest that these effects are due to the specificity of learning principle, in that acquiring skills under an internal focus prepares individuals for the self-focus conditions that are induced as a result of the presence of anxiety. Finally, results demonstrated that if learners are given no explicit focus instructions during learning they are susceptible to choking under anxious conditions. As such, coaches should endeavor to either minimize the acquisition of explicit movement knowledge during the learning process by encouraging an external focus of attention, or apply the principles of specificity to prevent choking by instructing participants to adopt a self-focus during long periods of learning.

## Conflict of Interest Statement

The authors declare that the research was conducted in the absence of any commercial or financial relationships that could be construed as a potential conflict of interest.

## Footnotes

^1^Initially 30 participants volunteered to partake in the investigation. However, one participant dropped out of the internal focus of attention group during testing due to random reasons. As such, the investigation was completed with unequal group sizes; *N* = 9 for the internal focus group, and 10 for both the external and control focus groups.

^2^Similar to Lawrence et al. (2011), it was decided that administering this manipulation check immediately following the LA transfer condition might act as a reminder of the focus instructions prior to the HA transfer condition.
